# Social overshadowing: Revisiting cue-competition in social interactions

**DOI:** 10.3758/s13423-022-02229-3

**Published:** 2023-01-05

**Authors:** Maïka Telga, José A. Alcalá, Cecilia Heyes, Gonzalo P. Urcelay

**Affiliations:** 1 School of Management, University of St Andrews, Gateway Building, North Haugh, St Andrews, KY16 9AJ UK; 2 University of Granada, Granada, Spain; 3 University of Leicester, Leicester, UK; 4 Complutense University of Madrid, Madrid, Spain; 5 School of Psychology, University of Nottingham, University Park, Nottingham, NG7 2RD UK; 6 University of Oxford, Oxford, UK

**Keywords:** Overshadowing, Learning, Stereotypes, Cooperation, Trust

## Abstract

In a large variety of contexts, it is essential to use the available information to extract patterns and behave accordingly. When it comes to social interactions for instance, the information gathered about interaction partners across multiple encounters (e.g., trustworthiness) is crucial in guiding one’s own behavior (e.g., approach the trustworthy and avoid the untrustworthy), a process akin to trial-by-trial learning. Building on associative learning and social cognition literatures, the present research adopts a domain-general approach to learning and explores whether the principles underlying associative learning also govern learning in social contexts. In particular, we examined whether overshadowing, a well-established cue-competition phenomenon, impacts learning of the cooperative behaviors of unfamiliar interaction partners. Across three experiments using an adaptation of the iterated Trust Game, we consistently observed a ‘social overshadowing’ effect, that is, a better learning about the cooperative tendencies of partners presented alone compared to those presented in a pair. This robust effect was not modulated by gender stereotypes or beliefs about the internal communication dynamics within a pair of partners. Drawing on these results, we argue that examining domain-general learning processes in social contexts is a useful approach to understanding human social cognition.

**Supplementary Information:**

The online version contains supplementary material available at 10.3758/s13423-022-02229-3.

Being able to make accurate inferences about others and behave accordingly is key for smooth and harmonious relationships. To navigate social interactions successfully, one often monitors others’ behavior across multiple encounters, infers their traits, and uses these inferences to decide how to act. For instance, repeated demonstrations of trustworthiness and cooperation over multiple encounters are likely responded to in a reciprocal fashion (King-Casas et al., 2005). Building on previous work adopting a reinforcement learning approach (Chang et al., 2010; Cho & Hackel, 2022; Fareri et al., 2015), the present research examined whether the framework of associative learning may contribute to our understanding of trait inferences and social interactions.

Research in associative learning has demonstrated that across multiple trials, human and nonhuman animals learn the reward value associated with stimuli and adjust their behavior accordingly, approaching rewarding stimuli and avoiding nonrewarding stimuli (Bouton, 2007). A similar approach may account for human behaviors in social contexts: across repeated interactions, people may learn social values such as a person’s generosity or trustworthiness, and based on this learning decide how to act whilst interacting with this person (Hackel et al., 2015). If learning of social values followed the same principles as learning in non-social contexts, the basic phenomena of associative learning would be expected in the social realm, as suggested by theories that underline the fundamental importance of domain-general, taxon-general processes in human cognition (Heyes, 2019; Lockwood et al., 2020; Reader, 2016; van Overwalle, 2011). In the present research, we test this hypothesis and examine whether cue-competition, a hallmark of associative learning, also characterizes learning about the cooperative tendencies of unfamiliar interaction partners.

Cue-competition comprises a family of phenomena that underly learning about the relationships between events, for example a cue and an outcome, when such learning occurs in the presence of multiple cues. Among cue-competition phenomena, blocking and overshadowing have been investigated extensively. Blocking is the phenomenon in which learning about the reward value of a target cue X is impaired when trained in the presence of a cue A, which receives additional training either before (forward-blocking) or after (backward-blocking) XA compound training (Kamin, 1968; Shanks, 1985). Similarly, overshadowing refers to impaired learning about a cue X that was trained in compound with a cue B, compared to when trained alone (Pavlov, 1927). Blocking and overshadowing have been observed, not only in nonhuman animals, but in studies of human contingency judgment (Price & Yates, 1993; Shanks, 1985), Pavlovian conditioning (Martin & Levey, 1991), spatial learning (Herrera et al., 2022), or evaluative conditioning (Kattner & Green, 2015). All these examples suggest that cue-competition is due to domain-general mechanisms that are present in a wide range of species (Urcelay, 2017).

However, only a few studies have explored cue-competition in social contexts, and these have focused on blocking (Cramer et al., 1985; FeldmanHall et al., 2017; Mata et al., 2021). As far as we are aware, there have been only two reports of ‘social overshadowing’ in humans, dating back to the 1980s. Lanzetta and Orr (1980, 1981) found that social cues, such as faces varying in emotional expression, differentially impact learning about a tone-shock relationship in a fear conditioning paradigm: when a face expressing either fear or happiness and a neutral stimulus (i.e., tone) were trained in compound followed by a shock, fearful faces overshadowed the tone, and happy faces were overshadowed by the tone. Hence, Lanzetta and Orr found asymmetrical learning about the cues within the compound (i.e., the tone and the face), depending on the emotion portrayed – a reciprocal overshadowing effect suggesting that social information (faces) was being processed associatively with asocial information (tone). However, they did not include a control cue trained alone, leaving unanswered the question of whether learning about the target predictor (e.g., face) was overshadowed or potentiated by the presence of a second predictor (e.g., tone). In the current study, we aimed to overcome the limitations of previous overshadowing research in social contexts by examining trait inferences through social interactions in a classic overshadowing design with appropriate controls. Moreover, we aim to explore the potential interaction of learning processes with social mechanisms related to group membership such as stereotypes and intergroup biases.

To achieve this goal, we used an adaptation of the iterated Trust Game (King-Casas et al., 2005), which has been used extensively to explore trial-by-trial learning (e.g., Meidinger & Terracol, 2012), and the expression of social biases (e.g., Slonim & Guillen, 2010; Telga et al., 2018). The Trust Game recreates cooperation dynamics between people that resemble associative learning, as participants learn to approach rewarding stimuli (i.e., cooperative partners) and to avoid nonrewarding stimuli (i.e., noncooperative partners) on a trial-by-trial basis. Moreover, it allows us to explore potential social biases by manipulating partners’ features such as their social category membership. Across three experiments, we used the Trust Game to examine overshadowing in social interactions and its potential modulation by gender stereotypes (Experiments 1–3) and participants’ knowledge of the communication dynamics among partners (Experiment 3).

In Experiment 1, we used the Trust Game as a learning phase in which participants learned the cooperative (or uncooperative) tendencies of unfamiliar male and female game partners, presented either alone or in a pair (with a counterpart of the same gender). Next, in a test phase, participants were asked to indicate their likelihood of cooperating with each one of the partners from the Trust Game, now presented alone. We anticipated an overshadowing effect, that is, more learning of the cooperative tendencies of partners presented alone compared to those presented within a pair. Moreover, because women are associated with more cooperation than men (Buchan et al., 2008; Telga et al., 2018), we also assessed whether partners’ gender would impact the magnitude of the overshadowing effect, such that stereotype-inconsistent associations would be more vulnerable to competition, resulting in more overshadowing for male compared to female partners.

In Experiment 2, we used a similar design but introduced a new condition to explore whether the stereotypical belief that females are more trustworthy than males (Buchan et al., 2008) could modulate overshadowing. Like Lanzetta and Orr (1980, 1981), who observed that fearful faces overshadowed neutral stimuli in fear conditioning, we examined whether female faces would overshadow male faces when learning trustworthiness. For this, we introduced an additional mixed-gender pair (i.e., one male and one female partner within a pair) to the design.

Overshadowing is typically explained by associative processes (Mackintosh, 1976; Pearce, 1987) but the social overshadowing effects observed in Experiments 1 and 2 could instead have been due to reasoning (de Houwer et al., 2005). Participants may have learned less about their partner when the partner appeared in a pair because the participants inferred that the decision to cooperate or not was influenced by the other member of the pair, and therefore provided less information about the partner’s trustworthiness. This is a potential explanation because, when pairs of partners were presented in Experiments 1 and 2, it was ambiguous whether the two members of the pair consensually decided how to act, or one member of the pair made a unilateral decision. We tested this alternative explanation in Experiment 3 by replicating Experiment 1 with an additional between-participants manipulation of instructions. In the non-ambiguous group, participants were instructed that partners presented in a pair made consensual decisions, hence their final decision reflected the cooperative tendency of both members. In the ambiguous group, ambiguity was similar to Experiments 1 and 2. We expected that if reasoning was responsible for the overshadowing effect, it should be replicated in the ambiguous group, but eliminated in the non-ambiguous group.

## Methods

### Participants

Sample size (a minimum of 40 participants per group) was based on previous research using the Trust Game (Telga et al., 2018). Participants were recruited through Prolific online crowdsourced platform and received a fixed financial compensation for their time (i.e., a minimum of £8.55/h), plus a bonus proportional to their performance (i.e., £0.011 per pound earned in the Trust Game), for a minimum of £2.85 and a maximum of £6 for the entire experiment. We recruited 48 participants in Experiment 1 (24 women, *M*_*age*_ = 38.0 years, *SD*_*age*_ = 16.4) and Experiment 2 (24 women, *M*_*age*_ = 34.7 years, *SD*_*age*_ = 12.2), and 96 participants (48 women, *M*_*age*_ = 37.2 years, *SD*_*age*_ = 13.0) in Experiment 3. A sensitivity analysis using G*Power (Faul et al., 2007) showed that with this sample, we could detect an effect of ${\eta}_{\text{p}}^{\text{2}}$ = .15 (β = .80, α = .05) for the critical Partner Behavior × Trial Type interaction reflecting an overshadowing effect. Note that in all experiments, the effect observed was larger than .15. To control for the impact of cultural background on cooperation decisions (Gächter et al., 2010), we pre-screened participants to select only those who were born, had spent most of their childhood, and were currently living in the same country – the United Kingdom. We also pre-screened participants who had already taken part in studies of this experimental series. Consent was obtained at the beginning of the experiments. All the studies were approved by the University of Leicester ethics committee (ref: 27997).

### Stimuli and materials

Gorilla software (Anwyl-Irvine et al., 2020) was used for stimulus presentation and data collection. All the materials used in these experiments are available on the Gorilla Open Materials Repository (https://app.gorilla.sc/openmaterials/451374). Sixteen colored photographs were extracted from the Chicago Face Database (Ma et al., 2015) to represent the game partners (six men and six women in Experiments 1 and 3, eight men and eight women in Experiment 2). All faces were presented against a white background with a direct gaze and a neutral emotional expression. The Chicago Face Database is a standardized set of faces including ratings about the perceived sex, race, or trustworthiness of all targets. This information was used to select the faces to be used in the Trust Game. Specifically, we ensured that all faces belonged to the same racial category (i.e., all White), and were approximately the same age (*M* = 27.6 years, *SD* = 1.6 in Experiments 1 and 3; *M* = 27.1 years, *SD* = 1.5 in Experiment 2). We also ensured that there was no difference between male and female partners regarding their age, *F*(1,10) = .02, *p* = .88, ${\eta}_{\textrm{p}}^2$ < .01, in Experiments 1 and 3, *F*(1, 14) = .38, *p* = .55, ${\eta}_{\textrm{p}}^2$ = .03, in Experiment 2, and their perceived trustworthiness, *F*(1,10) = .60, *p* =.46, ${\eta}_{\textrm{p}}^2$ = .06, in Experiments 1 and 3, *F*(1, 14) = .12, *p* = .73, ${\eta}_{\textrm{p}}^2$ < .01 in Experiment 2.

### Procedure

After providing consent, participants started the experiment, which comprised three phases: baseline, learning, and test. The complete instructions provided to the participants are provided in Sections 1 and 2 of the Online Supplementary Materials (OSM).

First, the baseline allowed us to assess participants’ spontaneous likelihood to cooperate with each one of the partners before the Trust Game. Each trial consisted of a single display in which the photograph of one partner was presented in the center of the screen and the question “*How likely are you to spontaneously cooperate with this person?*” was displayed below the photograph. To respond, participants used a horizontal slider ranging from 0 (Very Unlikely) to 100 (Very Likely). Once they had indicated their ratings, participants moved to the next trial by pressing the “*Continue*” button. Although there was no time limit, participants were encouraged to respond as fast as possible. The order of presentation of the faces was randomized for each participant.

**Fig. 1:**
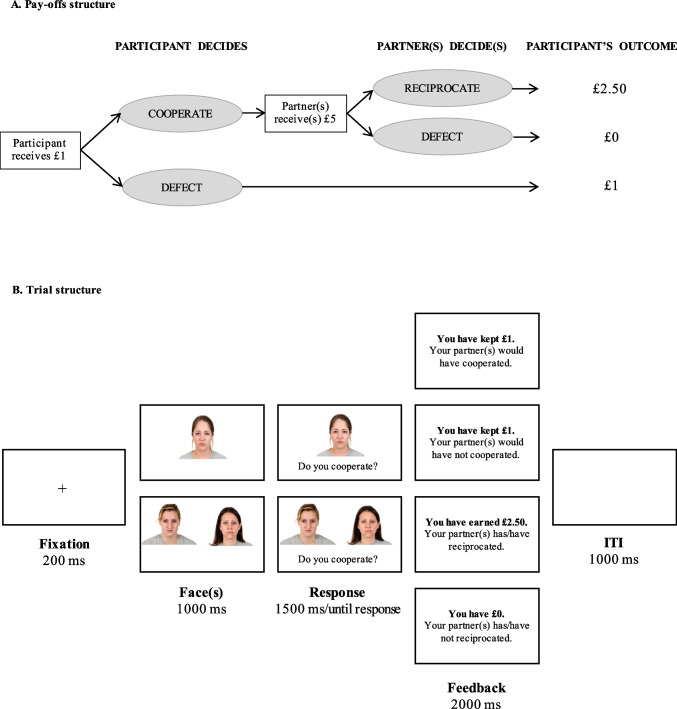
Pay-off structure (**a**) and timeline of one trial (**b**) in the Trust Game

Next, participants performed the learning phase, consisting of an adaptation of the Trust Game (Berg et al., 1995; Telga et al., 2018), as shown in Fig. 1. At the beginning of each trial, participants virtually received £1 and had to decide what to do with it. After a fixation point appeared in the center of the screen for 200 ms, participants saw the image of the partner or pair of partners of this round for 1,000 ms. Next, the question “*Do you cooperate?*” appeared below the photograph(s). Participants had 1,500 ms to indicate whether they cooperated by pressing the ‘1’ key or did not cooperate by pressing the ‘0’ key. If participants decided to cooperate, the partner or pair of partners in this round would ostensibly receive the initial amount multiplied by 5 (i.e., £5) and decide whether to either give £2.50 back to the participant (i.e., reciprocate) or to keep all the money (i.e., defect). In this case, they were presented a 2,000-ms feedback display informing of their partner(s) decision (i.e., either reciprocated or defected) together with their final monetary outcome (i.e., either £2.50 or £0). If, in contrast, participants decided not to cooperate, they would keep the initial £1 and the partner(s) would receive nothing. In that case, they were presented a 2,000-ms feedback screen informing of their monetary outcome (i.e., £1) as well as the decision that the partner would have made if they had cooperated (i.e., the partner could have either reciprocated or defected). Trials were separated by a 1,000-ms inter-trial interval (ITI).

Before they started the Trust Game, participants received detailed instructions about the task, specifying the payoff structure of a trial. Next, they performed an eight-trial practice with four partners (different from those presented in the Trust Game). For these practice trials, participants were encouraged to alternately cooperate and not cooperate, so they could become familiarized with the four different types of feedback. They were also told that their responses in the practice were not considered to compute their final earnings. Upon completion of the practice trials, participants started the Trust Game in which we manipulated their partners’ gender, partners’ behavior, and the type of trials.

In Experiment 1, we manipulated within-participants the cooperative behaviors of 12 different partners: six men and six women. These partners were presented either individually (single partner condition) or in a pair (pair of partners condition). Partners presented in a pair were of the same gender (i.e., either two men or two women) and always displayed with the same counterpart across trials. Whether partners presented in a pair were displayed on the left or on the right side of the screen was counterbalanced across trials. Within these two trial type conditions, half of the partners were always cooperative and allowed participants to earn money (i.e., cooperative partners condition) while the other half of partners were always noncooperative, leading participants to no monetary gain (i.e., noncooperative partners condition). The eight types of trials resulting from the factorial combination of partners’ gender (female vs. male), trial type (single vs. pair), and partners’ behavior (cooperative vs. noncooperative) were presented 20 times, resulting in 160 training trials.

Experiment 2 used a similar design, except that two mixed-gender pairs (one cooperative pair, and one noncooperative pair) were added to the aforementioned design. Therefore, participants were presented with 16 different partners, eight men and eight women, for a total of ten types of trials presented 20 times, resulting in 200 trials.

Finally, Experiment 3 used a similar design to Experiment 1, with an additional between-group manipulation of the instructions provided to participants. Specifically, in the non-ambiguous group, participants were told that when a pair of partners decided to cooperate (defect), both of them had agreed and therefore both were cooperative (noncooperative). In the ambiguous group, participants were told that when a pair of partners made a decision to cooperate (defect), it meant that either one of them or both of them made the final call, and therefore either one of them or both were cooperative (noncooperative). After the instructions, we introduced a manipulation check to ensure that participants knew how decisions were made within a pair. Participants who failed this check re-experienced the instructions until they correctly responded to all questions.

In all experiments, the order of presentation of the trials was pseudo-randomized within ten mini-blocks of two presentations of each type of trial. We also ensured that across participants, all the photographs of the same gender were alternately associated with the four experimental conditions resulting from the combination of trial type and partner behavior. Participants had two opportunities to take a short break, up to 1 min, after Block 3 and after Block 7.

The test phase was similar to the baseline phase, except that participants were asked to evaluate the likelihood that they would cooperate with each partner on the basis of what they had learned during the learning phase, rather than based on their first impression. The experiment took around 20 min to complete.

### Data exclusion

Participants who responded on fewer than 20% of the trials in the learning phase were excluded from the analyses. Following this criterion, we eliminated two participants from the analyses of Experiment 2, leaving 46 participants. In Experiments 1 and 3, data from all participants were included in the analyses.

### Design and analyses

The baseline allowed us to obtain an initial score of participants’ likelihood to cooperate with each partner. This DV was analyzed as a function of partner gender (female vs. male), while controlling for participant gender (female vs. male). With this design, a cooperation bias toward one of the two genders should be reflected in a main effect of partner gender. We expected a higher likelihood to cooperate with female than with male partners (Buchan et al., 2008; Telga et al., 2018).

For the learning phase in Experiment 1, we analyzed participants’ cooperation rate (i.e., the proportion of trials on which they cooperated with their partner(s)) as a function of partner gender (female vs. male), partner behavior (cooperative vs. noncooperative), trial type (single vs. pair), and block (1–10). In Experiment 2, the same DV was analyzed as a function of partner behavior (cooperative vs. noncooperative), trial type (female single, male single, female pair, male pair, mixed-gender pair), and block (1–10). In Experiment 3, we used the same design as in Experiment 1, with an additional between-participants variable of ambiguity. In all experiments, we controlled for participants’ gender by introducing this variable in the ANOVA. The analyses of the learning phase of all experiments are reported in Section 3 of the OSM.

For the test phase, we subtracted the likelihood to cooperate with each partner in the baseline phase from the likelihood to cooperate with them in the test phase, to control for potential spontaneous biases in cooperation. This DV was analyzed as a function of partner gender (male vs. female), partner behavior (cooperative vs. noncooperative), and trial type (single vs. same-gender pair (vs. mixed-gender pair in Experiment 2)). In Experiment 3, we added the between-participants variable ambiguity (ambiguous vs. non-ambiguous) to the aforementioned design. Overall, we expected an increase in the likelihood to cooperate with cooperative partners, and a decrease in the likelihood to cooperate with noncooperative partners. Critically, with this design, an overshadowing effect should be reflected in a significant Partner Behavior × Trial Type interaction, indicating a larger increase in the likelihood to cooperate with cooperative partners presented alone compared to those presented in a pair, and a larger decrease in the likelihood to cooperate with noncooperative partners presented alone compared to those presented in a pair.

## Results

### Baseline phase

A perusal of Table 1 suggests that in all experiments, participants were consistently more likely to cooperate with female than with male partners. In fact, a mixed-design ANOVA with participant gender (female vs. male) as a between-participants factor and partner gender as a within-participants variable revealed that the main effect of partner gender was significant in Experiment 1, *F*(1, 46) = 8.18, *p* < .01, ${\eta}_{\textrm{p}}^2$ = .15, 90% CI [.03, .30], Experiment 2, *F*(1, 44) = 9.06, *p* < .01, ${\eta}_{\textrm{p}}^2$ = .17, 90% CI [.03, .33], and Experiment 3, *F*(1, 94) = 7.24, *p* < .01, ${\eta}_{\textrm{p}}^2$ = .07, 90% CI [.01, .17]. These effects were not qualified by participant gender in Experiment 1, *F*(1, 46) = 2.22, *p* = .14, ${\eta}_{\textrm{p}}^2$ = .05, 90% CI [.00, .17], Experiment 2, *F*(1, 44) = 0.16, *p* = .70, ${\eta}_{\textrm{p}}^2$ < .01, 90% CI [.00, .08], or Experiment 3, *F*(1, 94) = 0.07, *p* = .79, ${\eta}_{\textrm{p}}^2$ < .01, 90% CI [.00, .03].

**Table 1:** Descriptive statistics for the baseline phase

	Female partners	Male partners
Experiment 1	55.9 (12.7)	50.5 (11.3)
Experiment 2	53.8 (9.6)	49.0 (10.2)
Experiment 3	54.6 (12.3)	50.8 (11.2)

Means and standard deviations (in parentheses) of the likelihood to cooperate with female and male partners in the three experiments

### Test phase

**Fig. 2:**
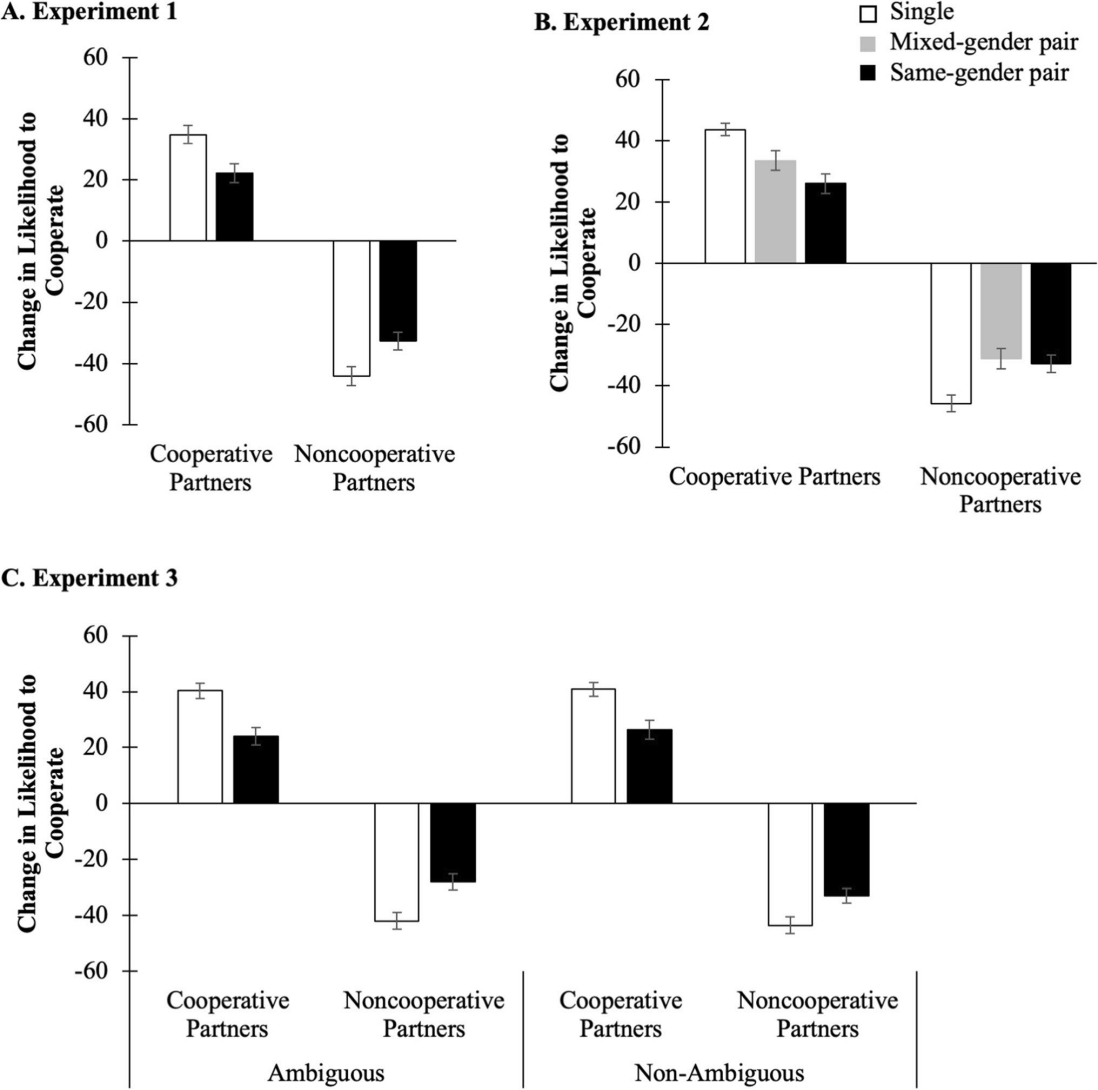
Change in the likelihood to cooperate from the baseline to the test phase in Experiment 1 (**a**), Experiment 2 (**b**), and Experiment 3 (**c**). *Note:* Error bars represent the standard error of the mean with within-participant correction following Cousineau and O’Brien (2014)

In all experiments, after interacting with cooperative and non-cooperative game partners, participants increased their likelihood to cooperate with cooperative partners, and decreased their likelihood to cooperate with non-cooperative partners. Critically, and as shown in Fig. 2, the tendency was more pronounced for partners presented alone compared to those presented in a same-gender or mixed-gender pair, consistent with the expected overshadowing effect. A mixed-design ANOVA with partner gender, partner behavior, and trial type as within-participants variables, and participant gender (and ambiguity in Experiment 3) as between-participants factors revealed that the critical Partner Behavior × Trial Type interaction was significant in Experiment 1, *F*(1, 46) = 33.43, *p* < .01, ${\eta}_{\textrm{p}}^2$ = .42, 90% CI [.23, .55], Experiment 2, *F*(2, 88) = 24.32, *p* < .01, ${\eta}_{\textrm{p}}^2$ = .36, 90% CI [.22, .46], and Experiment 3, *F*(1, 92) = 64.61, *p* < .01, ${\eta}_{\textrm{p}}^2$ = .41, 90% CI [.28, .51]. Moreover, this interaction was not qualified by partner gender or participant gender in Experiments 1–3, nor by ambiguity in Experiment 3.^1^ Inferential and Bayesian statistics of the nonsignificant interactions are provided in the OSM (Table 1). These revealed that overall, the data did not fit well with models including these interactions. We, therefore, analyzed the main effect of trial type in the cooperative and noncooperative partners conditions for each experiment.

In Experiment 1, and as shown in Fig. 2a, participants were more likely to cooperate with cooperative partners presented alone compared to those presented in a pair. Similarly, their likelihood to cooperate with noncooperative partners decreased more towards partners presented alone compared to those presented in a pair. In fact, the main effect of Trial Type was significant in both the cooperative, *F*(1, 47) = 19.06, *p* < .01, ${\eta}_{\textrm{p}}^2$ = .29, 90% CI [.12, .44], and the noncooperative, *F*(1, 47) = 17.81, *p* < .01, ${\eta}_{\textrm{p}}^2$ = .28, 90% CI [.11, .42], partners conditions.

In Experiment 2, in which we introduced a mixed-gender pair of partners, a similar pattern was observed. As shown in Fig. 2b, participants showed a larger increase in their likelihood to cooperate with cooperative partners presented alone compared to those presented in same-gender or mixed-gender pairs. The main effect of trial type was, in fact, significant in the cooperative partners condition, *F*(2, 90) = 15.12, *p* < .01, ${\eta}_{\textrm{p}}^2$ = .25, 90% CI [.12, .36], revealing significant differences in the increase in the likelihood to cooperate between the single and the same-gender conditions, *F*(1, 45) = 46.90, *p* < .01, ${\eta}_{\textrm{p}}^2$ = .51, 90% CI [.33, .62], and the single and the mixed-gender conditions, *F*(1, 45) = 10.71, *p* < .01, ${\eta}_{\textrm{p}}^2$ = .19, 90% CI [.05, .35], but not between same-gender and mixed-gender conditions, *F*(1, 45) = 3.78, *p* = .06, ${\eta}_{\textrm{p}}^2$ = .08, 90% CI [.00, .22]. Moreover, within the mixed-gender pair, the likelihood to cooperate with the female and the male partner did not significantly differ, *F*(1, 45) = 0.08, *p* = .79, ${\eta}_{\textrm{p}}^2$ < .01, 90% CI [.00, .06], with a BF_01_ = 4.56 supporting the absence of differences. As depicted in Fig. 2b, participants showed a similar pattern of behavior towards noncooperative partners. In fact, the main effect of trial type was also significant in the noncooperative partners condition, *F*(2, 90) = 11.03, *p* < .001, ${\eta}_{\textrm{p}}^2$ = .20, 90% CI [.08, .30], with significant differences between the single and the same-gender conditions, *F*(1, 45) = 17.90, *p* < .01, ${\eta}_{\textrm{p}}^2$ = .29, 90% CI [.11, .43], the single and the mixed-gender conditions, *F*(1, 45) = 20.27, *p* < .01, ${\eta}_{\textrm{p}}^2$ = .31, 90% CI [.13, .46], but not between the same-gender and the mixed-gender conditions, *F*(1, 45) = 0.18, *p* = .68, ${\eta}_{\textrm{p}}^2$ < .01, 90% CI [.00, .08]. Again, the decrease in likelihood to cooperate with the female or the male partners within the mixed-gender pair did not differ, *F*(1, 45) = 0.25, *p* = .62, ${\eta}_{\textrm{p}}^2$ < .01, 90% CI [.00, .09], with a BF_01_ = 4.10 supporting the absence of differences.

Finally, in Experiment 3, in which we manipulated between participants that decision-making within a pair was either ambiguous or non-ambiguous, a similar pattern was observed. As depicted in Fig. 2c, participants from the two groups showed a larger increase in their likelihood to cooperate with cooperative partners presented alone relative to cooperative partners presented in a pair. The same was observed in the decrease for non-cooperative partners, regardless of the ambiguity on how partners in a pair made the decision to either cooperate or not cooperate. The main effect of trial type was significant in both the cooperative, *F*(1, 95) = 46.54, *p* < .01, ${\eta}_{\textrm{p}}^2$ = .33, 90% CI [.20, .43], and the noncooperative, *F*(1, 95) = 34.66, *p* < .01, ${\eta}_{\textrm{p}}^2$ = .27, 90% CI [.15, .38], partners conditions.

## Discussion

Across three experiments, we investigated whether overshadowing characterizes learning about the cooperative tendencies of unfamiliar game partners. We found that participants learned more about the cooperative tendencies of partners presented alone compared to those presented in a pair, a result that was consistent across all experiments, and unaffected by gender category (Experiments 1, 2, and 3) and instructions (Experiment 3) manipulations.

The results of Experiment 3 suggest that the social overshadowing effects were due to associative processes rather than reasoning. If participants had inferred that a partner’s behavior provides less information about their cooperative tendencies when the partner appeared in a pair, because the other member of the pair may have influenced the partner’s decision, the overshadowing effect should have disappeared in the non-ambiguous group. In fact, there was no difference between the non-ambiguous and the ambiguous groups. Across the three experiments, the overshadowing effect appeared in all eight conditions: when the partner was cooperative and non-cooperative, in a same- and mixed-gender pair, with ambiguous and non-ambiguous instructions. Although gender did not seem to interact with the overshadowing effect, we did observe a reliance on gender stereotypes at baseline with more cooperation with female than with male partners (Buchan et al., 2008; Telga & Lupiáñez, 2021), suggesting that this paradigm is sensitive to gender-related biases. Thus, the results of the present study suggest that models of associative learning (e.g., Rescorla & Wagner, 1972), developed in the nonsocial domain and predominantly in research with nonhuman animals, may advance our understanding of human social cognition (Behrens et al., 2008; Heyes, 2012), including the processes underlying cooperation and trust (FeldmanHall & Dunsmoor, 2018).

A priority for future research is to work out how domain-general processes such as associative learning and reasoning produce and interact with specifically social processes (Heyes, 2019; Heyes et al., 2020). Consistent with our results, a recent report has shown that social group membership does not modulate blocking in social learning (Vaz et al., 2022). However, there is also evidence that learning processes can interact with mechanisms specific to the social world. For instance, it has been shown that fear extinction, one of the most elemental associative learning phenomena, is modulated by stereotypical beliefs (Navarrete et al., 2009; Olsson et al., 2005). Similarly, using economic games, FeldmanHall et al. (2017) observed blocking in social decision making, but only in the gain rather than the loss domain, suggesting that specific social expectations may impact people’s reasoning about the likelihood that an event will occur (i.e., what are the odds for a person to steal or be altruistic). Moreover, the impact of social category information (e.g., gender stereotypes) largely depends on the salience of such categories (Freeman & Ambady, 2011) and the cognitive demands of the task (Gilbert & Hixon, 1991). This raises the possibility that more salient social categories, or a more complex learning task, would promote a larger reliance on stereotypical trait attribution, as observed in previous research (Telga et al., 2018). Future studies should aim to understand under which circumstances specific social mechanisms interact with domain-general processes to guide our behavior.

One of the challenges of studying learning processes in social contexts rests in recreating ecologically valid scenarios while maintaining methodological rigor. On that note, our adaptation of the Trust Game deviated from most real-life situations in that participants received feedback about their partners’ trustworthiness when they chose both to trust and not to trust them. This ensured that all participants started the test phase with the same exposure to their partners’ cooperative behaviors, so any observed effects are better explained by our experimental manipulations than by participants’ varying knowledge about their partners’ tendencies to cooperate. However, this may have resulted in a more accurate learning of others’ trustworthiness compared to what would be expected with an asymmetrical feedback design (Fetchenhauer & Dunning, 2010). Further research is needed to determine whether, with an asymmetrical feedback design and therefore poorer learning, the degraded contingency would attenuate the overshadowing effect (Urcelay, 2017).

The extension of research on cue-competition phenomena to social contexts opens interesting research avenues for both learning and social psychology theorists. The large body of research generated by the discovery of blocking and overshadowing has allowed researchers to propose prominent models that account for not only the phenomena themselves but also their neural substrates (Waelti et al., 2001), and their modulation by different factors (e.g., physical salience; Mackintosh, 1976). Thus, exploring cue-competition in social contexts offers to learning researchers a new area to test these theories and assess their generality and validity in the domain of complex social interactions. For instance, would social psychological salience impact overshadowing in a similar way as physical salience? Moreover, it offers a new perspective for social psychologists to explore phenomena such as impression formation and trait inferences. For instance, is the dynamism and malleability of person construal (Freeman & Ambady, 2011) subject to retrospective re-evaluation, as observed in backward blocking (Shanks, 1985)? A convergent approach to these phenomena, drawing on learning theory and social psychology, promises to provide a more comprehensive view of human social behavior.

Overall, the present research provides novel evidence that cue-competition may account for social behaviors such as trait inferences, trust, and cooperation. More broadly, it suggests that models of associative learning could be an asset in research on human social cognition.

## Supplementary information

ESM 1 (DOCX 7.69 mb)

## Footnotes

1 Note that to ensure that the absence of gender differences was not due to low statistical power, we analyzed the three experiments together (excluding the mixed-gender pair condition in Experiment 2). With this larger sample (*N* = 190), we observed the same pattern as observed in each experiment alone, that is, an overshadowing effect reflected in a significant Partner Behavior × Trial Type interaction, *F*(1, 184) = 133.83, *p* < .01, ${\eta}_{\textrm{p}}^2$ = .42, not qualified by Participant Gender, *F*(1, 184) = 0.89, *p* = .35, ${\eta}_{\textrm{p}}^2$ < .01, nor by any other variables, largest *F*(1, 184) = 1.39, *p* = .25, ${\eta}_{\textrm{p}}^2$ = .02, for the Partner Behavior × Trial Type × Partner Gender × Experiment interaction.
