# Supplementary material for: Social overshadowing: Revisiting cue-competition in social interactions
Source: Psychon Bull Rev. 2023 Jan 5;30(4):1575–85. doi: 10.3758/s13423-022-02229-3 (PMC10482779; doi:10.3758/s13423-022-02229-3)
Supplement: Supplementary file 1 — (DOCX 7.69 mb) [file 13423_2022_2229_MOESM1_ESM.docx]

Social Overshadowing: Revisiting cue-competition in social interactions

Maïka Telga^1,2,3^, José A. Alcalá^5^, Cecilia Heyes^4^ & Gonzalo P. Urcelay^5^

^1^ University of St Andrews

^2^ University of Granada

^3^ University of Leicester

^4^ University of Oxford

^5^ Complutense University of Madrid

# Supplementary Materials

## 1. Instructions Experiments 1 and 2

### 1.1. Instructions Baseline Phase

*Before you start with the trust game, you will be presented with the photographs of the people with whom you will play the game. Your task is to indicate what is the likelihood that you would* ***spontaneously*** *cooperate with each person from 0 (Very Unlikely) to 100 (Very Likely). Please, try to respond as fast as possible on the basis of your* ***spontaneous*** *impression about each person.*

### 1.2. Instruction Learning Phase

**[Display #1]**

***Phase 2: The Trust Game***

*Now, you are going to start the trust game. You are going to play with different partners in several rounds. In some rounds, you will play with a unique partner, and in some other rounds, you will play with a pair of partners. In both cases, at the beginning of each round, you will receive £1 and you will have to decide whether:*

- *You want to cooperate with your partner(s) in this round by sending them your £1, or*
- *You want to keep the £1 for yourself and do not want to cooperate.*

*If you decide to send your £1, your partner will receive £5. Then, your partner(s) will decide to share those £5 with you or not. You will be informed of your partner(s)’ decision in each round. In each round, your game partner(s) will decide between:*

- *Reciprocating, in this case they will give you back half of the money, £2.50, or*
- *Defecting, in this case they will keep the £5 and will give you nothing*

*If you decide not to cooperate and to keep the £1 at the beginning of the round, you will win this £1 and your game partner(s) will receive nothing in this round.*

*These are the possible outcomes in each round:*


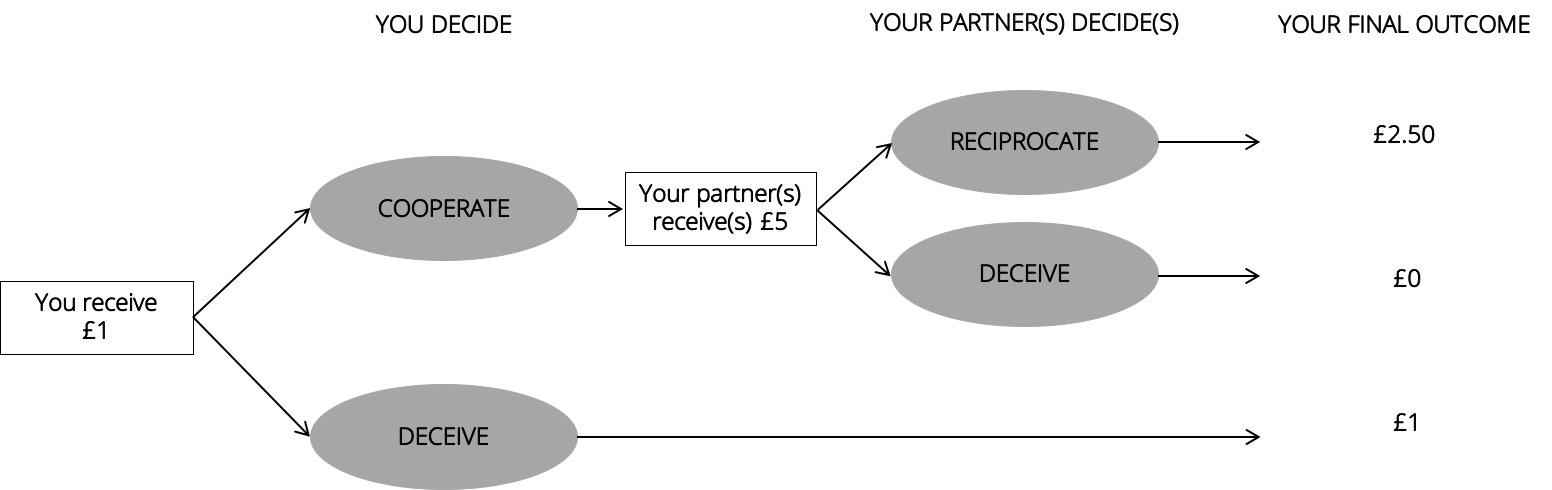


--

**[Display #2]**

***Your partners' decision.***

*These are the four possible feedbacks you will receive:*

*
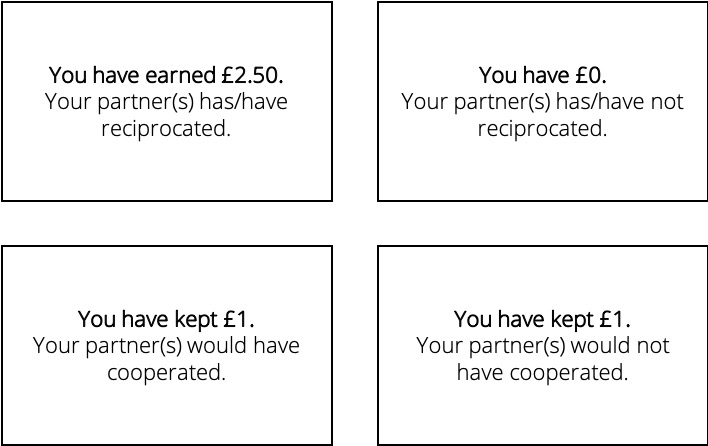
*

*Your goal is to maximize your benefits for the entire game. The best strategy to achieve this goal is to cooperate with partners who share the money with you, and to not cooperate with partners who do not to share the money with you. You will have several trials with all partners to determine their tendency to share.*

--

**[Display #3]**

***How to respond?***

*At the beginning of each round, you will receive £1. Next, a fixation point (+) will appear in the centre of the screen. Please focus on this point. Your partner(s) in this round will appear for 1 second and next, the question "Do you cooperate?" will appear for 1,5 seconds below the photograph(s) of your partner(s). Please, wait for the question to appear to make your decision to either cooperate or not cooperate. From the moment the question appears, you will have 1,5 seconds to respond. You must respond within this time.*

*If you decide to* ***SEND*** *your £1, press the* ***'1'*** *key.*

*If you decide to* ***KEEP*** *your £1, press the* ***'0'*** *key.*

*
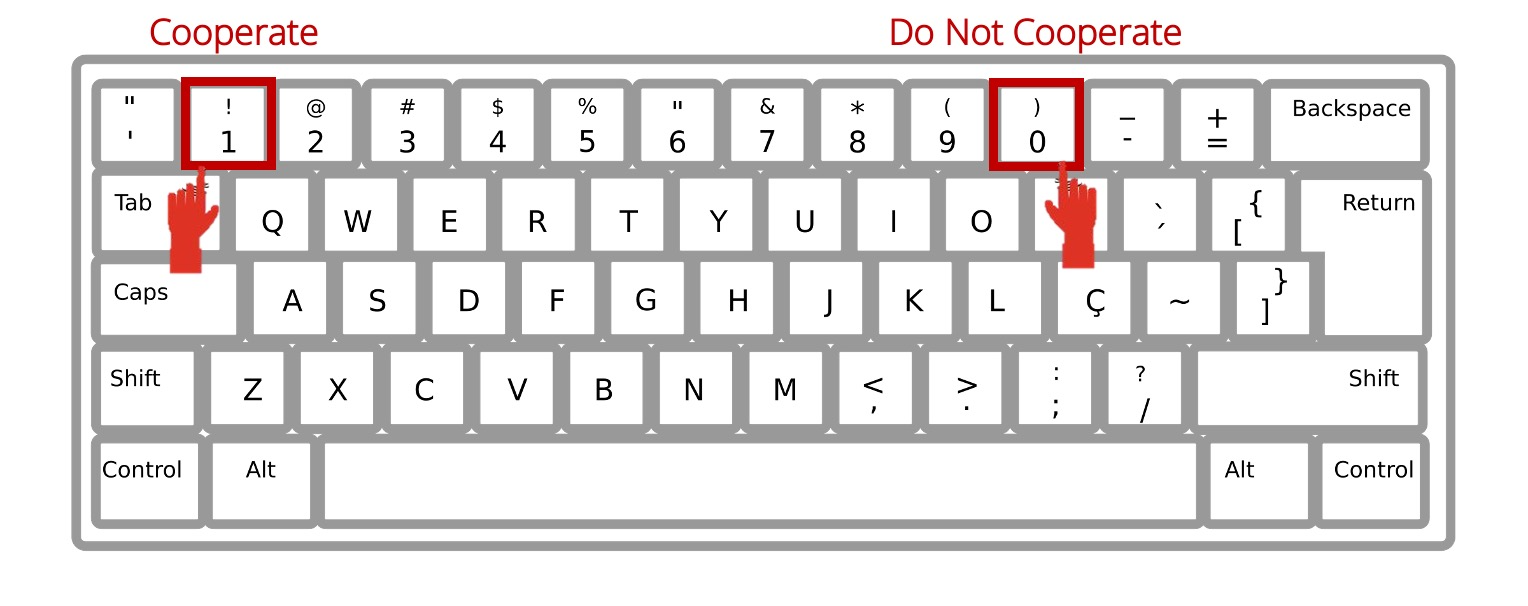
*

*After you decide, you will see the final result of this trial.*

--

**[Display #4]**

*Remember that your goal is to maximize your benefits with each partner. Also, note that you will only have 1,5 seconds to make your decision. In the case that you do not respond within these 1,5 seconds in more than 10% of the trials, you will not receive any extra monetary compensation.*

*Please note that the amount of money that is presented on each trial during this task is not the amount that you will receive upon completion of the study. You will earn £X.XX for participating in this study, independently of your performance, and will receive an extra compensation according to your performance, ranging from £0 to £X.XX. As a result, you are guaranteed to receive £X.XX upon completion of the study but may earn up to £6 depending on your performance. Our goal in presenting the specific amounts on each trial is to provide you with a simple example of how money can be earned and shared in this task.*

--

**[Display #5]**

***Practice Trials***

*Let’s start with a few practice trials. The following trials will help you to get familiarized with the trust game dynamic. Your partners in the practice trials are not the ones with whom you will play the trust game, and your outcomes in the practice trials will not affect your final monetary gains. Try to alternately cooperate and not cooperate, so you can see all the possible outcomes depending on your decision.*

--

**[Display #6]**

***Let's start the trust game!***

*Great! Now, let’s start the trust game. Remember, this is the payoffs structure of each trial:*

*
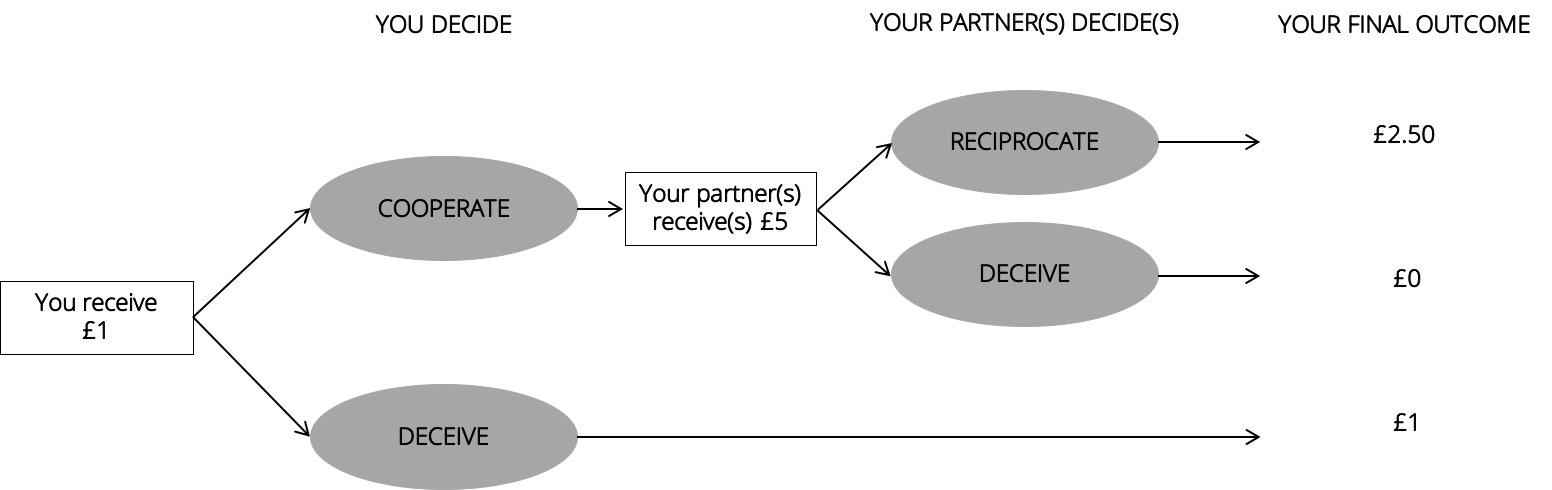
*

*Also, remember that you will only have 1,5 seconds to make your decision after the question "Do you cooperate?" appears. In the case that you last more than 1,5 seconds to respond in more than 10% of the trials, you will not receive any extra monetary compensation.*

### 1.3. Instructions Test Phase

*Now that you have played the trust game, you will be again presented with the photographs of the people with whom you played the game. Now, your task is to indicate what is the likelihood that you would cooperate with each person from 0 (Very Unlikely) to 100 (Very Likely)* ***on the basis of what you have learned during the game****. Please, try to respond as fast as possible,* ***keeping in mind your experience with each partner during the trust game****.*

## 2. Instructions Experiment 3

The instructions for Experiment 3 were identical to Experiments 1 and 2, except for the following display added on Display #2:

[Non-ambiguous group]

*People* ***in a pair will cooperate*** *with you only when each of them is cooperative. In other words, when a pair of partners cooperate with you, it means that* ***they are both cooperative****.*

*People in* ***a pair will not cooperate*** *with you only when each one of them is uncooperative. In other words, when a pair of partners does not cooperate with you, it means that* ***they are both uncooperative****.*

[Ambiguous group]

*People in* ***a pair will cooperate*** *with you when one of them is cooperative and when both of them are cooperative. In other words, when a pair of partners cooperate with you it means* ***either one or both of them is cooperative****.*

*People in* ***a pair will not cooperate*** *with you when one of them is uncooperative and when both or them are uncooperative. In other words, when a pair of partners does not cooperate with you, it means that* ***either one or both of them is uncooperative****.*

The manipulation checks at the end of the instructions were as follow:

*1. When does a pair of partners cooperate?*

*- When neither of them is cooperative*

*- When either one of them or both of them are cooperative*

*- When both of them are cooperative*

*2. What key should you use to cooperate?*

*- 0*

*- 1*

*- The spacebar*

*3. When does a pair of partners deceive (i.e., not cooperate)?*

*- When neither of them is uncooperative*

*- When either one of them or both of them are uncooperative*

*- When both of them are uncooperative*

## 3. Results Learning Phase

### 3.1. Experiment 1

To examine participants’ learning of the cooperative behaviors of their partners in the Trust Game, we conducted a mixed-design ANOVA on cooperation rates in the learning phase with partner gender (female vs. male), partner behavior (cooperative vs. noncooperative), trial type (single vs. pair) and block (1-10) as within-participants variables, and participant gender as a between-participants factor. The Partner Behavior x Block interaction indicating that participants learned to discriminate between cooperative and noncooperative partners across blocks was significant, *F*(9, 414) = 54.17, *p* < .01, $ƞ_{p}^{2}$ = .54, 90% CI [.48, .58], and qualified by Trial Type, *F*(9, 414) = 3.03, *p* < .01, $ƞ_{p}^{2}$ = .06, 90% CI [.01, .08], but not by partner gender *F*(9, 414) = 0.49, *p* = .88, $ƞ_{p}^{2}$ = .01, or participant gender, *F*(9, 414) = 1.33, *p* = .22, $ƞ_{p}^{2}$ = .03, 90% CI [.00, .04]. Moreover, the 5-way Participant Gender x Partner Gender x Partner Behavior x Trial Type x Block interaction was not significant, *F*(9, 414) = 0.99, *p* = .45, $ƞ_{p}^{2}$ = .02, 90% CI [.00, .03]. We therefore analyzed the Partner Behavior x Trial Type interaction in the last block of learning.

In Block 10, neither the main effect of Trial Type, *F*(1, 47) = 0.29, *p* = .59, $ƞ_{p}^{2}$ < .01, 90% CI [.00, .09], nor the Partner Behavior x Trial Type interaction were significant, *F*(1, 47) = 2.10, *p* = .15, $ƞ_{p}^{2}$ = .04, 90% CI [.00, .16]. The only significant effect was the main effect of Partner Behavior, *F*(1, 47) = 347.13, *p* < .01, $ƞ_{p}^{2}$ = .88, 90% CI [.82, .91], indicating that participants clearly discriminated between cooperative and noncooperative partners. Importantly, at the end of the learning phase, participants did not significantly differ in their cooperation between single (*M* = .90, *SD* = .22) and pairs (*M* = .88, *SD* = .26) of partners, *F*(1, 47) = 0.61, *p* = .44, $ƞ_{p}^{2}$ = .01, 90% CI [.00, .11], when they were cooperative. Neither did they significantly differ in their cooperation between single (*M* = .04, *SD* = .11) and pairs (*M* = .08, *SD* = .20) of partners, *F*(1, 47) = 1.81, *p* = .19, $ƞ_{p}^{2}$ = .04, 90% CI [.00, .16], when they were noncooperative.

### 3.2. Experiment 2

To examine participants’ learning of the cooperative behaviors of their partners in the Trust Game, we conducted a mixed-design ANOVA on cooperation rates in the learning phase with partner behavior (cooperative vs. noncooperative), trial type (female single, male single, female pair, male pair, mixed-gender pair) and block (1-10) as within-participants variables, and participant gender as a between-participants factor. The Partner Behavior x Block interaction indicating that participants learned to discriminate between cooperative and noncooperative partners across blocks was significant, *F*(9, 396) = 99.59, *p* < .01, $ƞ_{p}^{2}$ = .69, 90% CI [.65, .72], but not qualified by trial type, *F*(36, 1584) = 1.30, *p* = .11, $ƞ_{p}^{2}$ = .03, 90% CI [.00, .02], or participant gender, *F*(9, 396) = 0.77, *p* = .84, $ƞ_{p}^{2}$ = .02, 90% CI [.00, .02]. The 4-way Partner Gender x Partner Behavior x Trial Type x Block interaction was not significant either, *F*(36, 1584) = 1.29, *p* = .12, $ƞ_{p}^{2}$ = .03, 90% CI [.00, .03]. Importantly, at the end of the learning phase in Block 10, participants did not significantly differ in their cooperation between single, (*M* = .96, *SD* = .17), same-gender pair (*M* = .93, *SD* = .15), and mixed-gender pair (*M* = .91, *SD* = .26), *F*(2, 90) = 0.88, *p* = .42, $ƞ_{p}^{2}$ = .02, when they were cooperative. Similarly, they did not differ between single (*M* = .06, *SD* = .17), same-gender pair (*M* = .10, *SD* = .22), and mixed-gender pair (*M* = .05, *SD* = .16), when they were noncooperative, *F*(2, 90) = 1.17, *p* = .32, $ƞ_{p}^{2}$ = .02, 90% CI [.00, .09].

### 3.3. Experiment 3

To examine participants’ learning of the cooperative behaviors of their partners in the Trust Game, we conducted a mixed-design ANOVA on cooperation rates in the learning phase with partner gender (female vs. male), partner behavior (cooperative vs. noncooperative), trial type (single vs. pair) and block (1-10) as within-participants variables, and participant gender and ambiguity (ambiguous vs. non-ambiguous) as between-participants factor. The Partner Behavior x Block interaction indicating that participants learned to discriminate between cooperative and noncooperative partners across blocks was significant, *F*(9, 828) = 162.76, *p* < .01, $ƞ_{p}^{2}$ = .64, and not qualified by Ambiguity, *F*(9, 828) = 0.64, *p* = .77, $ƞ_{p}^{2}$ < .01. However, the Partner Gender x Partner Behavior x Trial Type x Block interaction was also significant, *F*(9, 828) = 3.28, *p* < .01, $ƞ_{p}^{2}$ = .03, although not qualified by Ambiguity, *F*(9, 828) = 0.66, *p* = .75, $ƞ_{p}^{2}$ < .01, 90% CI [.00, .01]. We therefore analyzed the last block of learning as a function of partner gender, partner behavior and trial type.

The three-way Partner Gender x Partner Behavior x Trial Type was not significant, *F*(1, 95) = 1.44, *p* = .23, $ƞ_{p}^{2}$ = .02, but the Partner Behavior x Trial Type was, *F*(1, 95) = 7.20, *p* = .01, $ƞ_{p}^{2}$ = .07. Simple effects analyses showed that for cooperative partners, participants cooperated more with partners presented alone (*M* = .96, *SD* = .14) compared to those presented in a pair (*M* = .90, *SD* = .23), *F*(1, 95) = 10.23, *p* < .01, $ƞ_{p}^{2}$ = .10. However, when partners were not cooperative, participants did not differ in their cooperation between partners presented alone (*M* = .05, *SD* = .17) compared to those presented in a pair (*M* = .08, *SD* = .18), *F*(1, 95) = 1.86, *p* = .18, $ƞ_{p}^{2}$ = .02, 90% CI [.00, .08].

## 4. Complementary Analyses: Test Phase (Experiment 3)

To ensure that this difference in the cooperative partners condition did not drive the overshadowing effect observed in the test phase, we further analyzed this condition by selecting only the participants who had learned to the same extent about partners presented alone and in a pair. We therefore conducted a mixed-design ANOVA on cooperation rates with cooperative partners in the test phase with Partner Gender (male vs. female) and Trial Type (single vs. pair) as within-participants variables, and Ambiguity and Participant Gender as between-participants factors. As expected, the main effect of trial type was significant showing that participants showed a larger increase in their likelihood to cooperate with partners presented alone (*M* = 41.43, *SD* = 17.77), compared to those presented in a pair (*M* = 31.24, *SD* = 19.13), *F*(1, 67) = 22.89, *p* < .01, $ƞ_{p}^{2}$ = .26, 90% CI [.11, .38]. This effect was not qualified by any other variables, largest *F*(1, 67) = 2.66, *p* = .11, $ƞ_{p}^{2}$ = .04, 90% CI [.00, .14], for the Partner Gender x Trial Type interaction.

## 5. Bayesian Analyses

Finally, we further examined the data with mixed-design Bayesian ANOVAs. Specifically, we computed Bayes factors inclusion (*BF_incl_*) across matched-models that quantifies the change from prior inclusion odds to posterior inclusion odds and can be interpreted as the evidence in the data for including one or several variables (see van den Bergh et al., 2020). For example, a *BF_incl_* of 5 for the critical Partner Behavior x Trial Type interaction indicates that the data are 5 times more likely under models that include this interaction than under models without it. More importantly, to better quantify the lack of effect on non-significant interactions, we computed Bayes factors exclusion (*BF_exc_*_l_) across matched-models that provide analogous information regarding the fit of the data under models that exclude specific predictors. In other words, a *BF_excl_* of 5 for the Partner Behavior x Trial Type x Participant Gender indicates that the data are 5 times more likely under models that exclude this interaction than under model that include it. Following the general guidelines for Bayesian analyses, we considered *BF* > 3 as substantial evidence in favor of the model tested (Jeffreys, 1961).

**Table 1**

*Inferential and Bayesian statistics of the critical interactions.*

| Exps | Interactions | Df | *F* | *p* | $ƞ_{p}^{2}$ | BF_exc_ | BF_incl_ |
| --- | --- | --- | --- | --- | --- | --- | --- |
| Exp1 | Participant Gender x Partner Gender x Partner Behavior x Trial Type | 1,46 | 0.12 | .73 | <.01 | 3.06 | 0.33 |
|  | Partner Gender x Partner Behavior x Trial Type | 1,46 | 0.07 | .80 | <.01 | 3.98 | 0.25 |
|  | Participant Gender x Partner Behavior x Trial Type | 1,46 | 2.85 | .10 | .06 | 1.85 | 0.54 |
|  | **Partner Behavior x Trial Type** | **1,46** | **33.43** | **<.01** | **.42** | **2.30e-4** | **4,341** |
|  |  |  |  |  |  |  |  |
| Exp2 | Participant Gender x Partner Gender x Partner Behavior x Trial Type | 2,88 | 0.56 | .57 | .01 | 4.74 | 0.01 |
|  | Partner Gender x Partner Behavior x Trial Type | 2,88 | 0.58 | .56 | .01 | 9.98 | 0.10 |
|  | Participant Gender x Partner Behavior x Trial Type | 2,88 | .18 | .84 | <.01 | 12.32 | 0.08 |
|  | **Partner Behavior x Trial Type** | **2,88** | **24.32** | **<.01** | **.36** | **4.51e-7** | **2.21e+6** |
|  |  |  |  |  |  |  |  |
| Exp3 | Ambiguity x Partner Gender x Partner Behavior x Trial Type | 1,92 | 0.49 | .49 | <.01 | 3.10 | 0.39 |
|  | Ambiguity x Partner Behavior x Trial Type | 1,92 | 0.51 | .48 | <.01 | 5.08 | 0.19 |
|  | Partner Gender x Partner Behavior x Trial Type | 1,92 | 2.10 | .15 | .02 | 2.77 | 0.36 |
|  | **Partner Behavior x Trial Type** | **1,92** | **64.61** | **<.01** | **.41** | **7.48e-13** | **1.32e+12** |
